# Supplementary material for: Broad-high operating temperature range and enhanced energy storage performances in lead-free ferroelectrics
Source: Nat Commun. 2023 Sep 15;14:5725. doi: 10.1038/s41467-023-41494-1 (PMC10504284; doi:10.1038/s41467-023-41494-1)
Supplement: Supplementary file 1 — Supplementary Information [file 41467_2023_41494_MOESM1_ESM.pdf]

Supplementary Information

Broad-high operating temperature range and enhanced energy storage performances in lead-free ferroelectrics

Weichen Zhao[1], Diming Xu[1, *], Da Li[1], Max Avdeev[2], Hongmei Jing[3], Mengkang Xu[4], Yan Guo[1], Dier Shi[5], Tao Zhou[6], Wenfeng Liu[7], Dong Wang[8, *], Di Zhou[1, *]

[1] Electronic Materials Research Laboratory & Multifunctional Materials and Structures, Key Laboratory of the Ministry of Education & International Center for Dielectric Research, School of Electronic Science and Engineering, Xi'an Jiaotong University, Xi'an 710049, Shaanxi, China

[2] Australian Nuclear Science and Technology Organization, Lucas Heights, 2234, NSW, Australia

[3] School of Physics and Information Technology, Shaanxi Normal University, Xi'an 710062, Shaanxi, China

[4] State Key Laboratory for Strength and Vibration of Mechanical Structures, School of Aerospace, Xi'an Jiaotong University, Xi'an 710049, Shaanxi, China

[5] Department of Chemistry, Zhejiang University, Hangzhou 310027, PR China

[6] School of Electronic and Information Engineering, Hangzhou Dianzi University, Hangzhou 310018, Zhejiang, China

[7] State Key Laboratory of Electrical Insulation and Power Equipment, Xi'an Jiaotong University, Xi'an 710049, Shaanxi, China

[8] Frontier Institute of Science and Technology and State Key Laboratory for Mechanical Behaviour of Materials, Xi'an Jiaotong University, Xi'an 710049, Shaanxi China

* Corresponding Author.

E-mail addresses: diming.xu@xjtu.edu.cn (Diming Xu)

wang_dong1223@mail.xjtu.edu.cn (Dong Wang)

zhoudi1220@gmail.com (Di Zhou)

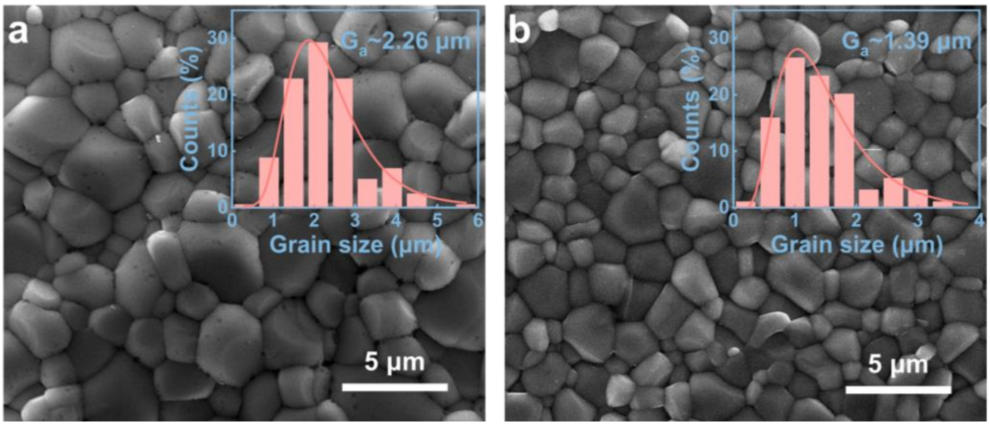

Supplementary Fig. 1 Natural surfaces SEM morphology and grain size distribution of the **a** BNKT-20SSN ceramic and **b** BNKT-20SSN (RRP) ceramic.

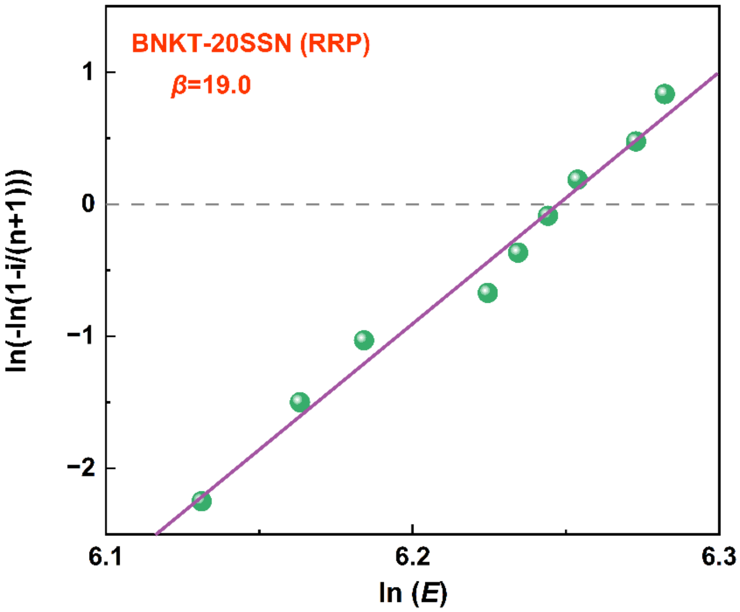

Supplementary Fig. 2 Weibull distribution of breakdown strength E_b for the BNKT-20SSN (RRP) ceramics.

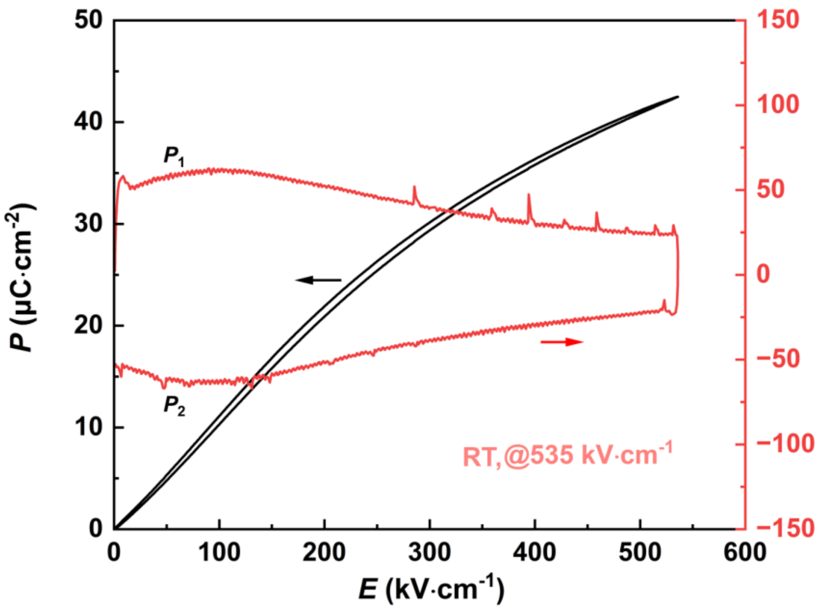

Supplementary Fig. 3 The unipolar *P-E* hysteresis loop and the *I-E* curve for the BNKT-20SS (RRP) ceramic.

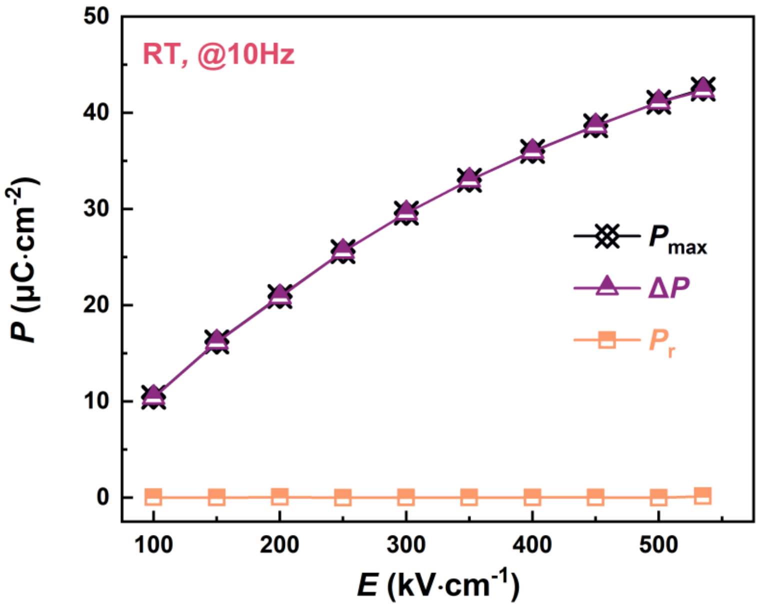

Supplementary Fig. 4 P_{max}, P_r, and ΔP values of the BNKT-20SSN (RRP) ceramic as a function of applied electric fields.

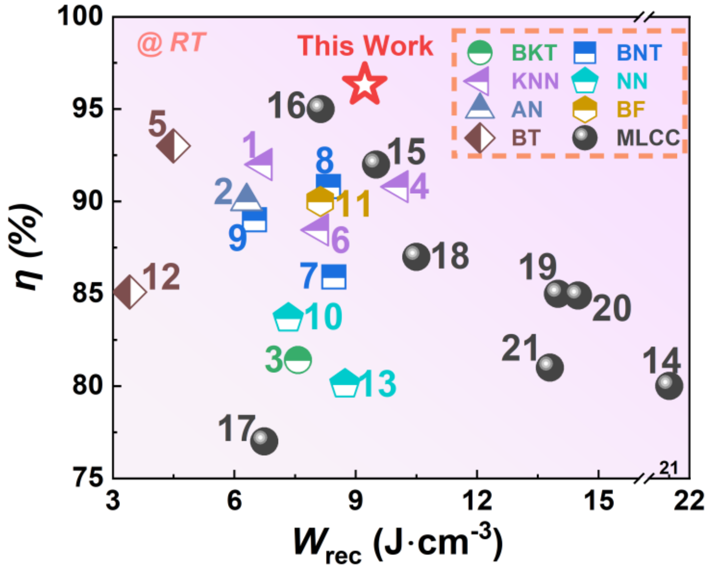

Supplementary Fig. 5. Comparisons of W_{rec} versus η (at room temperature) between our work with some recently reported lead-free bulk ceramics and certain MLCCs (Multi-Layer Ceramic Capacitors) [1-21].

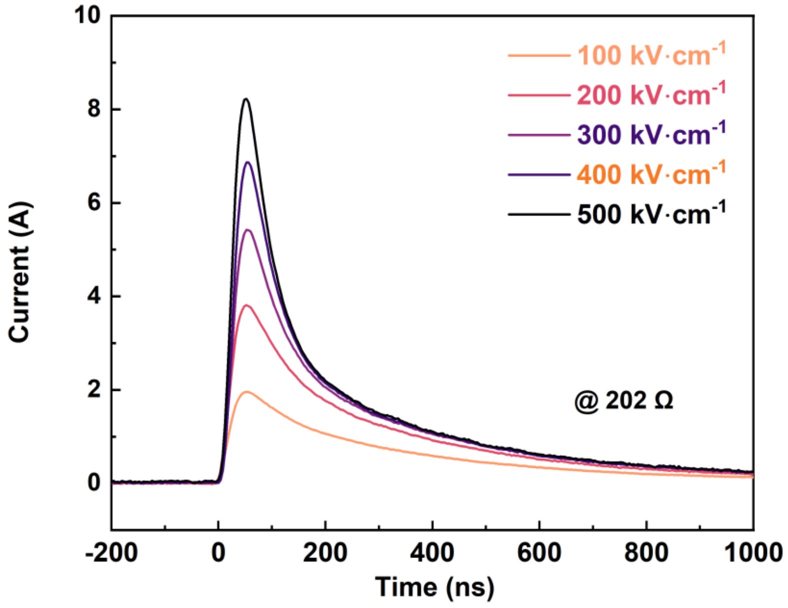

Supplementary Fig. 6 Overdamped discharging waveforms at various electric fields for the BNKT-20SSN ceramic (RRP).

+

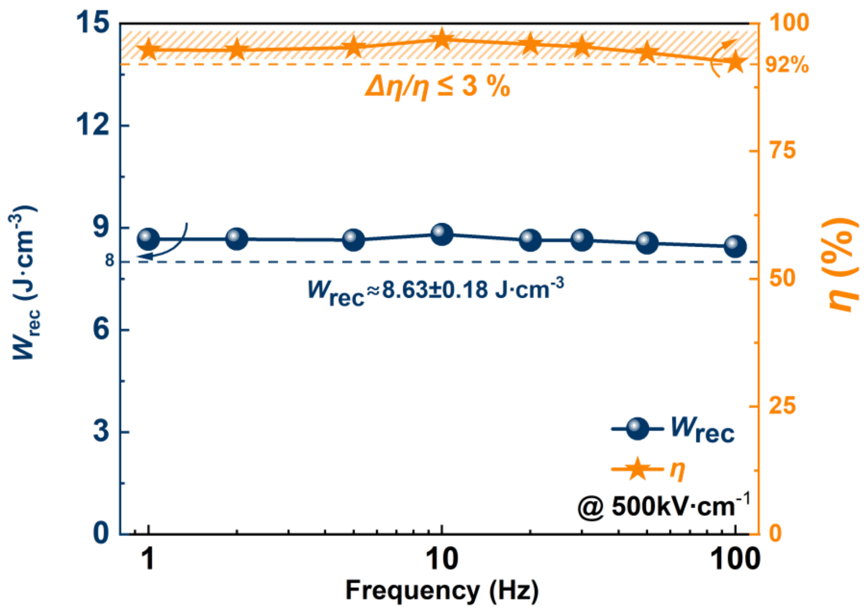

Supplementary Fig. 7 $W_{\rm rec}$ and η of the BNKT-20SSN ceramic (RRP) as a function of frequency under 500 kV cm^{-1}.

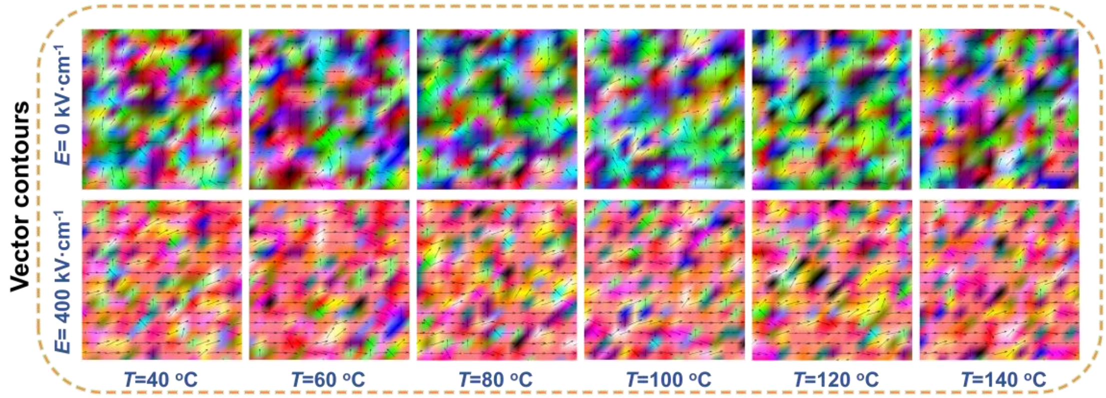

Supplementary Fig. 8 Vector contour of the BNKT-20SSN ceramic microstructure evolution generated by phase field simulation at various temperatures under 0 and 400 kV·cm^{-1}, respectively.

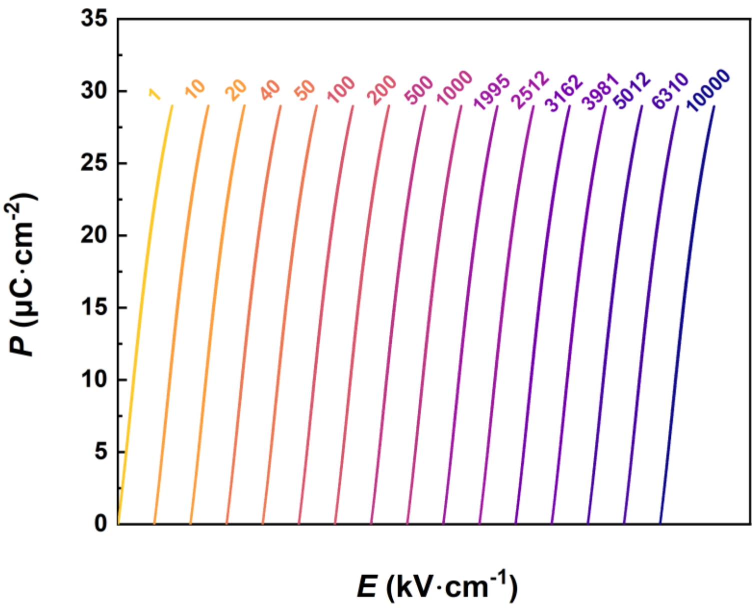

Supplementary Fig. 9 Unipolar *P-E* hysteresis loops of the BNKT-20SSN ceramic (RRP) as a function of the cycling numbers ($1\text{-}10^4$) under 300 kV cm^{-1}.

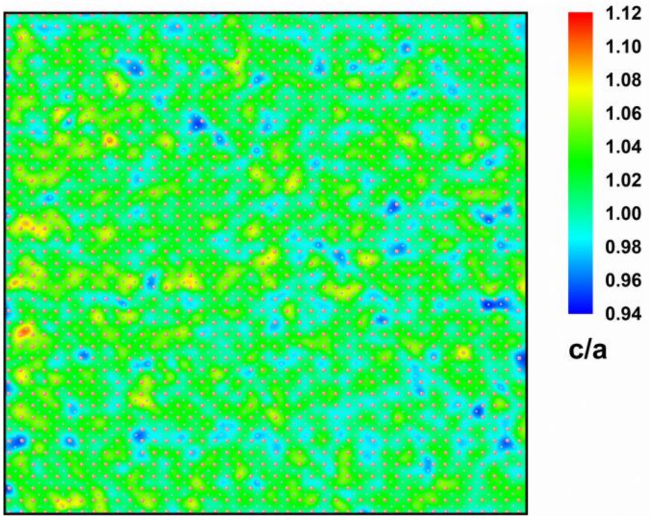

Supplementary Fig. 10 Axial ratio (c/a) mapping of BNKT-20SSN, obtained by calculating the corresponding atomic positions.

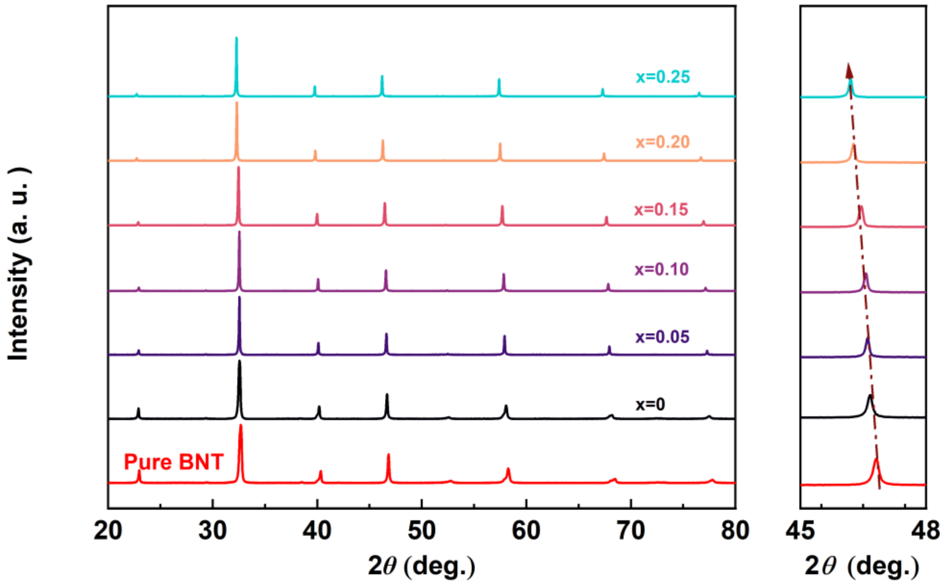

Supplementary Fig. 11 PXRD patterns of pure BNT and (1-*x*)BNKT-*x*SSN ceramics. By enlarging the PXRD patterns over the *2θ* range of 45-48° of each component, as is shown in Supplementary Fig. 11b, it is obvious that the diffraction peaks gradually shift to lower *2θ* angles with the increase of SSN content, which implies that the increase of cell parameters. This phenomenon may be due to the substitution of Bi^{3+} (R = 1.36 Å) and Na^{+}(R = 1.39 Å) by relatively large ionic radii of Sr^{2+} (R = 1.44 Å) on the A-site, as well as the ionic radius of Nb^{5+} (R = 0.64 Å) is larger than Ti^{4+} (R = 0.61 Å) on the B-site.

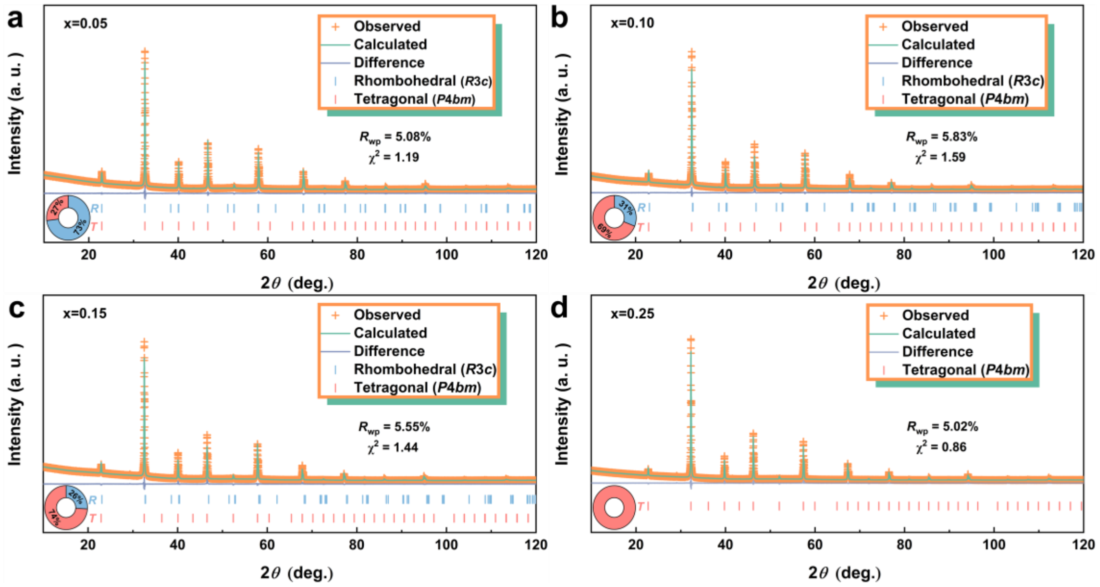

Supplementary Fig. 12 Rietveld refinement results of PXRD patterns for the (1-*x*)BNKT-*x*SSN ceramics: a x = 0.05, **b** x = 0.10, **c** x = 0.15, **d** x = 0.25. The R_{wp} (reliability factors of weighted patterns) and the χ^2 (goodness-of-fit indicator) of all components are reasonable, illustrating the refinement results are valid and the analysis of phase structure is credible [22].

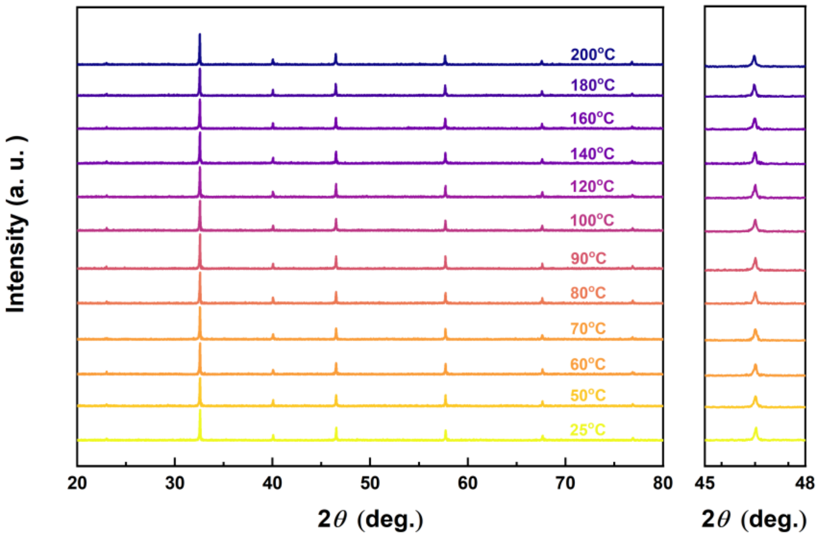

Supplementary Fig. 13 Temperature-dependent PXRD patterns of the BNKT-20SSN ceramic from 25 to 200 °C.

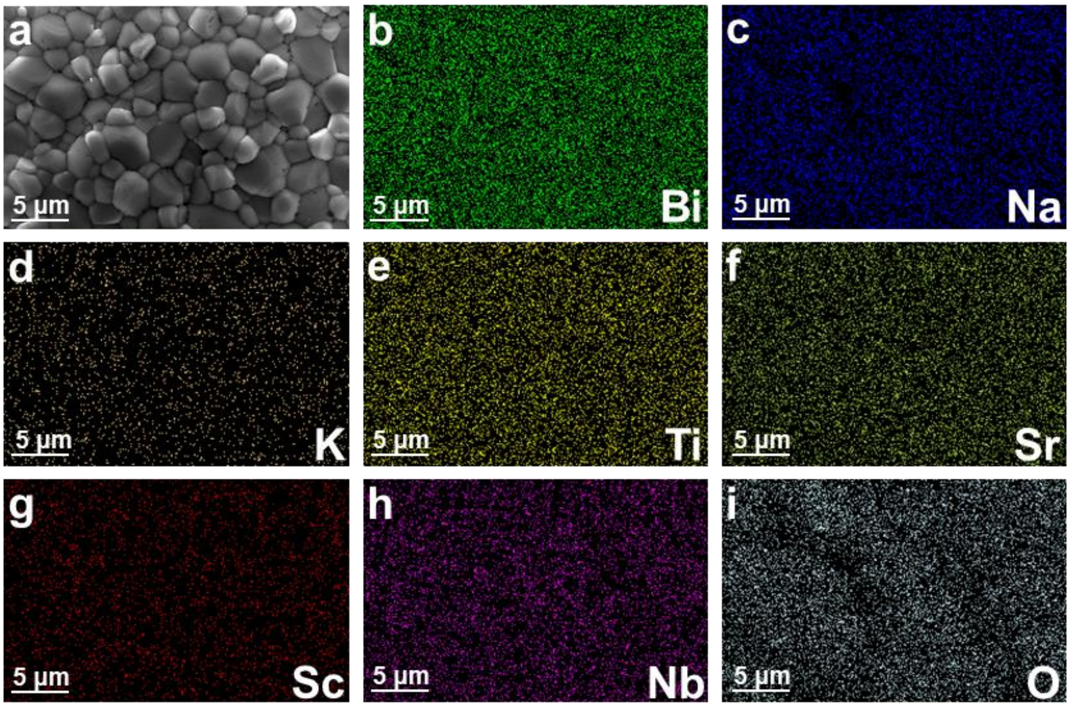

Supplementary Fig. 14 The SEM micrograph and element distribution maps of BNKT-20SSN ceramic. a SEM micrograph and **b-i** Energy-dispersive X-ray spectrometry element-mapping images. All of the elements exhibit homogeneous distribution characteristics with no segregation.

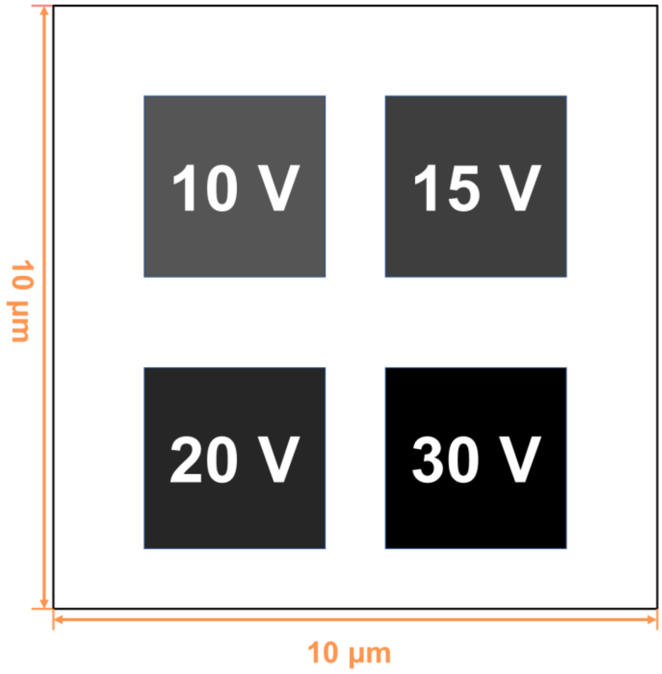

Supplementary Fig. 15 Schematic diagram of the applied voltages in the PFM test.

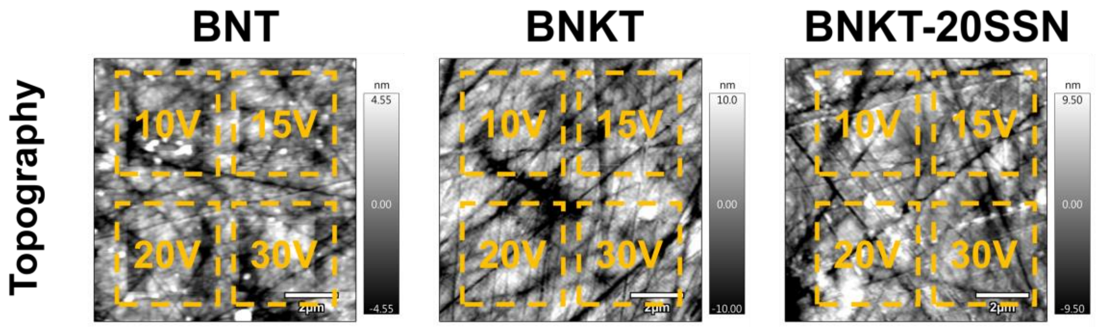

Supplementary Fig. 16 Topography images of the PFM samples.

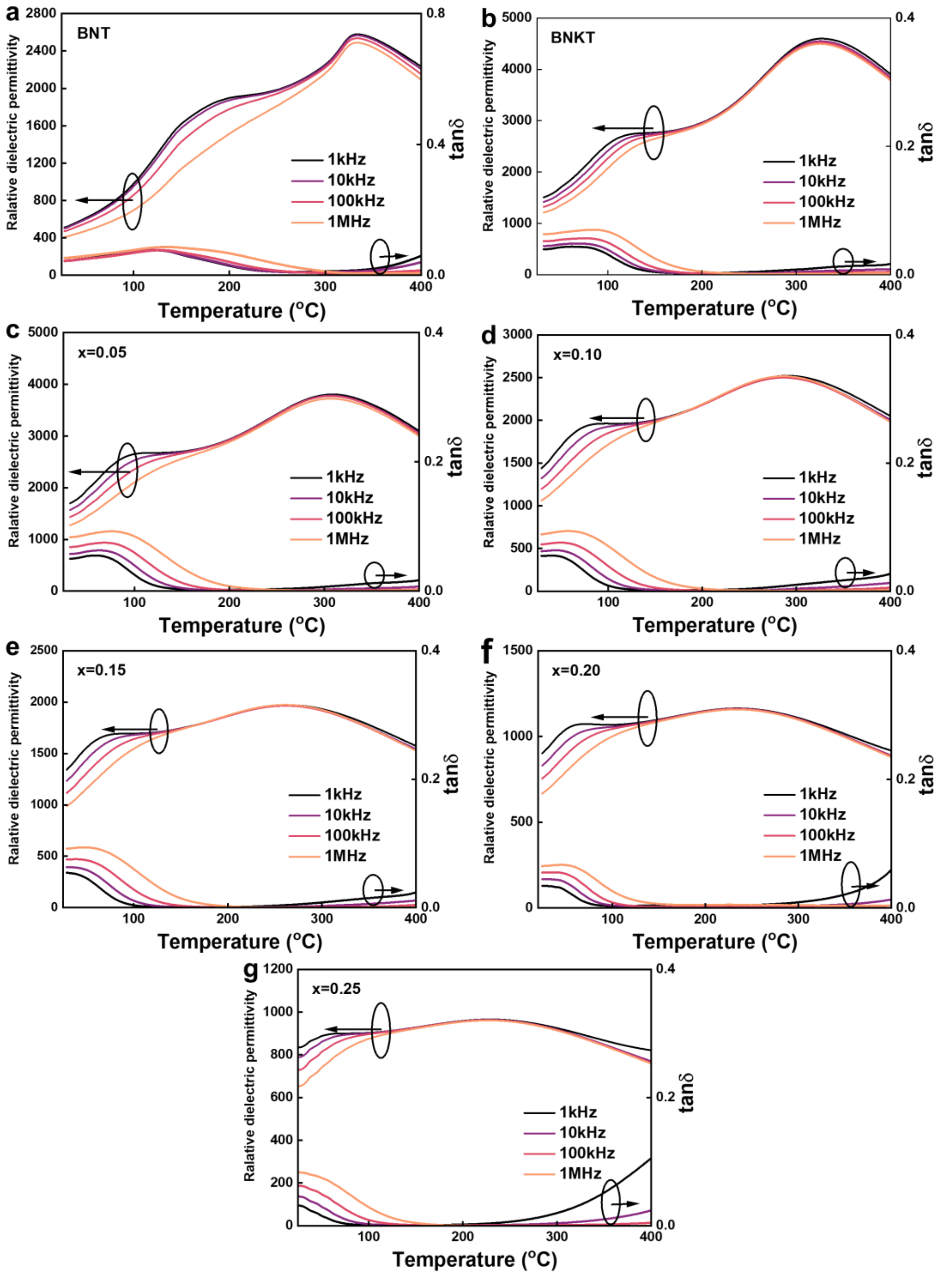

Supplementary Fig 17 Temperature-dependent dielectric permittivity and tanδ with different frequencies for the **a** BNT and (1-x)BNKT-xSSN ceramics: **b** $x = 0$, **c** $x = 0.05$, **d** $x = 0.10$, **e** $x = 0.15$, **f** $x = 0.20$, **g** $x = 0.25$.

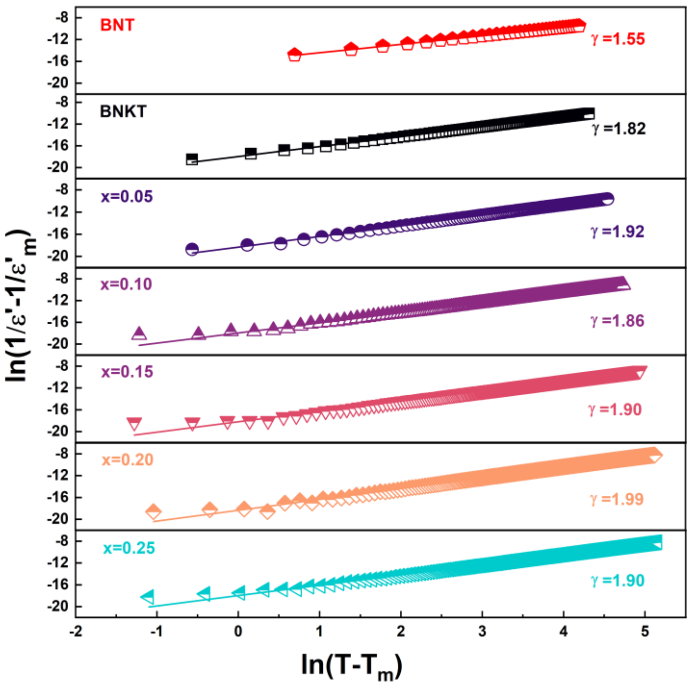

Supplementary Fig 18 ln(1/ε'-1/ε_m') versus ln(T-T_m) for BNT and (1-x)BNKT-xSSN ceramics at 1MHz.

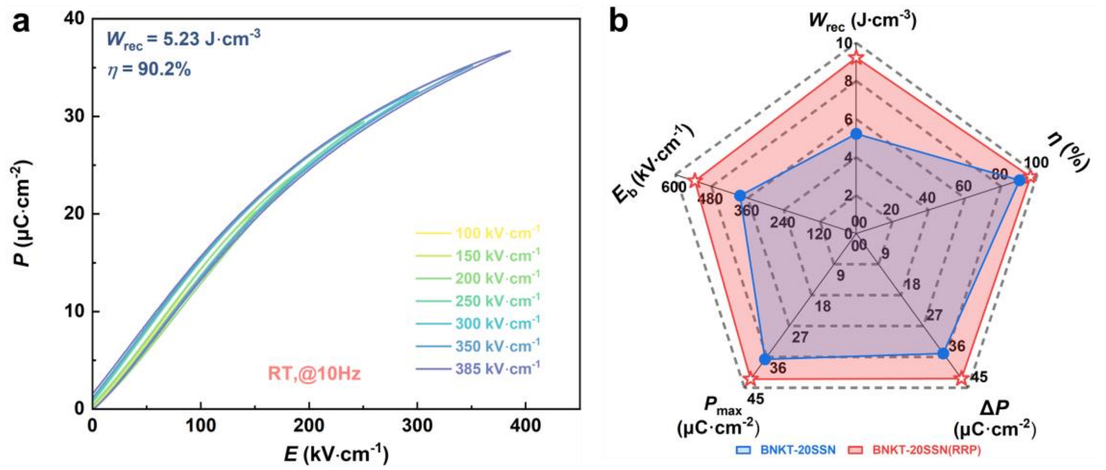

Supplementary Fig. 19 The energy storage performances of BNKT-20SSN ceramic. **a** Unipolar P-E hysteresis loop measured till the maximum applied electric fields. **b** Comparisons of comprehensive properties (W_{rec}, E_b, P_{max}, ΔP, η) between the BNKT-20SSN ceramic and BNKT-20SSN (RRP) ceramic. The BNKT-20SSN ceramic (RRP) covers the majority of the radar chart, suggesting a significant improvement in energy storage overall performance.

Supplementary Table 1

A comparison of the W_{rec} versus η between the BNKT-20SSN ceramic (RRP) with some recently reported lead-free bulk ceramics and certain MLCCs.

Compositions	W_{rec} (J·cm^{-3})		η (%)		Ref.
	RT	~150 ℃	RT	~150 ℃	
**Lead-free bulk ceramics**					
$0.85K_{0.5}Na_{0.5}NbO_3$-$0.15Bi(Zn_{2/3}Ta_{1/3})O_3$	6.7	3.0	92.0	68.2	1
$0.45AgNbO_3$–$0.55AgTaO_3$	6.3	4.2	90.0	89.7	2
$0.60Bi_{0.5}K_{0.5}TiO_3$-$0.30BaTiO_3$-0.10 $NaNbO_3$	7.6	4.4 $_{130\ ℃}$	81.4	89.2 $_{130\ ℃}$	3
$K_{0.5}Na_{0.5}NbO_3$-H	10.1	3.3 $_{140\ ℃}$	90.8	79.7 $_{140\ ℃}$	4
$BaTiO_3$-$Bi(Mg_{1/2}Ti_{1/2})O_3$	4.5	4.0	93.0	93.5	5
$0.85K_{0.5}Na_{0.5}NbO_3$-$0.15Bi(Ni_{0.5}Zr_{0.5})O_3$	8.1	1.9 $_{140\ ℃}$	88.5	82.3 $_{140\ ℃}$	6
$0.90(Bi_{0.5}Na_{0.5})_{0.65}Sr_{0.35}TiO_3$-$0.10Bi(Mg_{0.5}Zr_{0.5})O_3$	8.5	5.1 $_{140\ ℃}$	85.9	86.3 $_{140\ ℃}$	7
0.94 $Bi_{0.5}Na_{0.5}TiO_3$-$0.06BaTiO_3$-$0.15Sr(Al_{0.5}Ta_{0.5})O_3$	8.3	4.8	90.8	90.3	8
$0.80Bi_{0.5}Na_{0.5}TiO_3$-$0.20Sr(Nb_{0.5}Al_{0.5})O_3$	6.5	3.3	89.0	93.8	9
$Na_{0.7}Bi_{0.1}(Nb_{0.9}Ta_{0.1})O_3$	7.3	2.8 $_{120\ ℃}$	83.7	77.1 $_{120\ ℃}$	10
$0.57BiFeO_3$-$0.33BaTiO_3$-$0.10NaNbO_3$	8.1	4.7	90.0	92.1	11
$0.90BaTiO_3$-$0.10Bi(Mg_{0.5}Zr_{0.5})O_3$	3.4	2.8	85.1	88.2	12
$0.68NaNbO_3$-$0.32Bi_{0.5}Li_{0.5}TiO_3$	8.7	5.7	80.1	76.8	13
**MLCCs**					
$0.65Na_{0.5}Bi_{0.5}TiO_3$-$0.35Sr_{0.7}Bi_{0.2}TiO_3$ (<111>-textured)	21.5	16.6	80.0	81.2	14
$0.55Na_{0.5}Bi_{0.5}TiO_3$-$0.45Sr_{0.7}Bi_{0.2}TiO_3$	9.5	5.0 $_{120\ ℃}$	92.0	85.2 $_{120\ ℃}$	15
$0.87BaTiO_3$-$0.13Bi(Zn_{2/3}(Nb_{0.85}Ta_{0.15})_{1/3})O_3$	8.1	4.9	95.0	93.5	16
$0.75Bi_{0.85}Nd_{0.15}FeO_3$-$0.25BaTiO_3$	6.7	3.6 $_{125℃}$	77.0	68.1 $_{125\ ℃}$	17
$0.62BiFeO_3$-$0.30BaTiO_3$-$0.08Nd(Zn_{0.5}Zr_{0.5})O_3$	10.5	2.4	87.0	63.9	18
$Sm_{0.05}Ag_{0.85}(Nb_{0.7}Ta_{0.3})O_3$	14.0	7.0 $_{120\ ℃}$	85.0	94.5 $_{120\ ℃}$	19
$0.40(Bi_{0.5}Na_{0.5}TiO_3)$-$0.60(0.87BaTiO_3$-$0.13Bi(Zn_{2/3}(Nb_{0.85}Ta_{0.15})_{1/3})O_3)$	14.5	4.6	84.9	89.3	20
$0.57BiFeO_3$-$0.30BaTiO_3$-$0.13Bi(Li_{0.5}Nb_{0.5})O_3$	13.8	4.7 $_{100\ ℃}$	81.0	66.0 $_{100\ ℃}$	21
**BNKT-20SSN ceramic (RRP)**					
**$0.80Bi_{0.5}(Na_{0.82}K_{0.18})_{0.5}TiO_3$-$0.20Sr(Sc_{0.5}Nb_{0.5})O_3$**	**9.2**	**8.2**	**96.3**	**95.0**	**This work**

Supplementary Table 2

Refinement results of BNKT-20SSN derived from NPD refinement.

**NPD Refinement Results**		
**Space group**	***P4bm***	***R3c***
a / Å	5.5498(4)	5.5542(3)
b / Å	5.5498(4)	5.5542(3)
c / Å	3.9279(5)	13.5140(3)
V / Å^3	120.981(8)	361.04(2)
R_{wp}	5.12%	
χ^2	2.87	

**Atomic Parameters**				
P4bm				
**Atom**	**x**	**y**	**z**	**U_{iso}/10^3 Å^3**
Na1	0.00000	0.50000	0.5100(1)	0.0619(3)
Bi2	0.00000	0.50000	0.5100(1)	0.0619(3)
Ti3	0.00000	0.00000	0.012(9)	0.0400
O4	0.00000	0.00000	0.4685(3)	0.032(5)
O5	0.2592(5)	0.2408(5)	-0.021(6)	0.0281(2)
K6	0.00000	0.50000	0.5100(1)	0.0619(3)
Sr7	0.00000	0.50000	0.5100(1)	0.0619(3)
Sc8	0.00000	0.00000	0.012(9)	0.0400
Nb9	0.00000	0.00000	0.012(9)	0.0400
R3c				
**Atom**	**x**	**y**	**z**	**U_{iso}/10^3 Å^3**
Na1	0.00000	0.00000	2.548(5)	0.0619(3)
Bi2	0.00000	0.00000	2.548(5)	0.0619(3)
Ti3	0.00000	0.00000	10.279(2)	0.0400
O4	0.142(1)	0.337(2)	0.362(4)	0.0184(2)
K5	0.00000	0.00000	2.548(5)	0.0619(3)
Sr6	0.00000	0.00000	2.548(5)	0.0619(3)
Sc7	0.00000	0.00000	10.279(2)	0.0400
Nb8	0.00000	0.00000	10.279(2)	0.0400

Supplementary References

1. Li, D. et al. Improved energy storage properties achieved in (K, Na)NbO_3-based relaxor ferroelectric ceramics via a combinatorial optimization strategy. *Adv. Funct. Mater.* **32**, 2111776 (2021).
2. Luo, N. et al. Constructing phase boundary in $AgNbO_3$ antiferroelectrics: pathway simultaneously achieving high energy density and efficiency. *Nat. Commun.* **11**, 4824 (2020).
3. Chen, L. et al. Outstanding energy storage performance in high-hardness $(Bi_{0.5}K_{0.5})TiO_3$-based lead-free relaxors via multi-scale synergistic design. *Adv. Funct. Mater.* **32,** 2110478 (2021).
4. Chen, L. et al. Giant energy-storage density with ultrahigh efficiency in lead-free relaxors via high-entropy design. *Nat. Commun.* **13**, 3089 (2022).
5. Hu, Q. et al. Achieve ultrahigh energy storage performance in $BaTiO_3$-$Bi(Mg_{1/2}Ti_{1/2})O_3$ relaxor ferroelectric ceramics via nano-scale polarization mismatch and reconstruction. *Nano Energy* **67**, 104264 (2020).
6. Zhang, M. et al. Significant increase in comprehensive energy storage performance of potassium sodium niobate-based ceramics via synergistic optimization strategy. *Energy Storage Mater*. **45**, 861-868 (2022).
7. Zhu, X. et al. Ultrahigh energy storage density in $(Bi_{0.5}Na_{0.5})_{0.65}Sr_{0.35}TiO_3$-based lead-free relaxor ceramics with excellent temperature stability. *Nano Energy* **98** 107276 (2022).
8. Li, D. et al. Lead-Free relaxor ferroelectric ceramics with ultrahigh energy storage

densities via polymorphic polar nanoregions design. *Small* **19**, 2206958 (2022).

9. Yan, F. et al. Gradient-structured ceramics with high energy storage performance and excellent stability. *Small* **19**, 2206125 (2023).
10. Yang, W. et al. Superior energy storage properties in $NaNbO_3$-based ceramics via synergistically optimizing domain and band structures. *J. Mater. Chem. A* **10**, 11613-11624 (2022).
11. Qi, H. et al. Superior energy-storage capacitors with simultaneously giant energy density and efficiency using nanodomain engineered $BiFeO_3$-$BaTiO_3$-$NaNbO_3$ lead-free bulk ferroelectrics. *Adv. Energy. Mater.* **10,** 1903338 (2019).
12. Yuan, Q. et al. Bioinspired hierarchically structured all-inorganic nanocomposites with significantly improved capacitive performance. *Adv. Funct. Mater.* **30**, 2000191 (2020).
13. Xie, A. et al. $NaNbO_3$-$(Bi_{0.5}Li_{0.5})TiO_3$ lead-free relaxor ferroelectric capacitors with superior energy-storage performances via multiple synergistic design. *Adv. Energy. Mater.* **11**, 2101378 (2021).
14. Li, J. et al. Grain-orientation-engineered multilayer ceramic capacitors for energy storage applications. *Nat. Mater.* **19**, 999-1005 (2020).
15. Li, J. et al. Multilayer lead-free ceramic capacitors with ultrahigh energy density and efficiency. *Adv. Mater.* **30**, 1802155 (2018).
16. Cai, Z. et al. High-temperature lead-free multilayer ceramic capacitors with ultrahigh energy density and efficiency fabricated via two-step sintering. *J. Mater. Chem. A* **7**, 14575-14582 (2019).

17. Wang, D. et al. Bismuth ferrite-based lead-free ceramics and multilayers with high recoverable energy density. *J. Mater. Chem. A* **6**, 4133-4144 (2018).

18. Wang, G. et al. Ultrahigh energy storage density lead-free multilayers by controlled electrical homogeneity. *Energy Environ. Sci.* **12**, 582-588 (2019).

19. Zhu, L.F. et al. Heterovalent-doping-enabled atom-displacement fluctuation leads to ultrahigh energy-storage density in $AgNbO_3$-based multilayer capacitors. *Nat. Commun.* **14**, 1166 (2023).

20. Zhao, P. et al. Ultrahigh energy density with excellent thermal stability in lead-free multilayer ceramic capacitors via composite strategy design. *J. Mater. Chem. A* **9**, 25914-25921 (2021).

21. Wang, G. et al. Fatigue resistant lead-free multilayer ceramic capacitors with ultrahigh energy density. *J. Mater. Chem. A* **8**, 11414-11423 (2020).

22. Toby, B. et al. GSAS-II: the genesis of a modern open-source all purpose crystallography software package. *J. Appl. Cryst.* **46**, 544-549 (2013).
